# WGM microprobe device for high-sensitivity ultrasound detection and vibration spectrum measurement

**DOI:** 10.1007/s12200-025-00161-7

**Published:** 2025-08-14

**Authors:** Jialve Sun, Shengnan Huangfu, Tinglan Chen, Zijing Cai, Bowen Ruan, Fangxing Zhang

**Affiliations:** 1 Key Laboratory for Advanced Optoelectronic Integrated Chips of Jiangsu Province, Peking University Yangtze Delta Institute of Optoelectronics, Nantong, 226010 China; 2 School of Physics, Peking University, Beijing, 100871 China; 3 Department of Electronic Engineering, Tsinghua University, Beijing, 100084 China

**Keywords:** Whispering-gallery-mode (WGM) microprobe, Ultrasound detection, Photoacoustic imaging, Vibration spectroscopy

## Abstract

Whispering-gallery-mode (WGM) microcavities have emerged as a promising alternative to traditional ultrasound probes, offering high sensitivity and wide bandwidth. In our research, we propose a novel silica WGM microprobe device, with impressive *Q* factors up to 10^7^. The side-coupled approach and special encapsulation design make the device compact, robust, and capable of utilizing in both gaseous and liquid environments. We have successfully conducted photoacoustic (PA) imaging on various samples using this device which demonstrates a high sensitivity of 5.4 mPa/√Hz and a broad bandwidth of 41 MHz at −6 dB for ultrasound. And it is capable of capturing the vibration spectrum of microparticles up to a few hundred megahertz. Our compact and lightweight device exhibits significant application potential in PA endoscopic detection, near-field ultrasound sensing and other aspects.

**Graphical Abstract:**

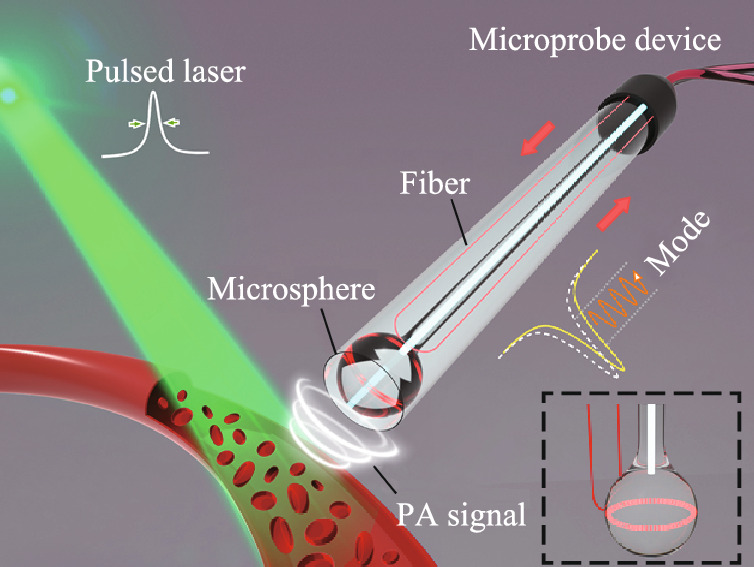

## Introduction

Ultrasound sensing finds widespread application across various domains, encompassing clinical ultrasound (US) imaging [1, 2], photoacoustic (PA) imaging [3, 4], and industrial non-destructive testing [5, 6], with the performance of the ultrasound detector being of paramount importance. In recent years, optical approaches for ultrasound detection, utilizing components such as microrings [7–9], Fabry–Pérot (FP) interferometers [10–12], and π-phase-shifted Bragg grating [13], have proved to outperform traditional piezoelectric-based ultrasound detectors in terms of both sensitivity and bandwidth [14]. Among those optical methods, whispering-gallery-mode (WGM)-based US detectors have emerged as a transformative platform due to their advantages, including but not limited to ultrahigh acoustic sensitivity, compact size for minimally invasive deployment, and all-optical architecture that eliminates electromagnetic interference [15, 16]. These advantages have driven their successful deployment in cellular and in vivo PA imaging [17, 18], vibrational spectroscopy detection [19], liquid sensing [20], and other diverse application domains [21, 22].

Although the WGM microcavity has superiority in its diminutive size of tens of micrometers, the practical dimensions of the reported WGM microcavity US detectors (including coupling fiber and supporting substrate) usually reaches several millimeters or even larger at the sensing probe head [13, 18, 23], posing challenges to either implementations within very tight space (such as vascular endoscopy) or performing near-field-like detection over targets with uneven surface. Therefore, a needle-like miniature ultrasound probe based on sensitive WGM microcavity is extremely desired [24]. As a highly sensitive platform, microcavities are also expected to be able to use beyond the laboratory with more subtle measurements. In addition, due to the large frequency span (MHz–GHz) and the ultrahigh sensitivity requirements of mesoscopic vibration spectroscopy, microcavity devices are highly valued as the most effective solution, albeit very challengingly.

In this work, we propose a side-coupled method of SiO_2_ microsphere to the folded fiber cone with controllable and robust encapsulation approach. Utilizing the spreadability and molecular surface tension of polymer microdroplets on the surface of silica microsphere, the tapered fiber is bound to the surface of the sphere for stable coupling with high *Q* factors. The sensor exhibits enhanced durability after fully encapsulation and the protection of the glass and metal tube. This innovative microprobe device boasts heightened ultrasound sensitivity and broad bandwidth, enabling the detection and imaging of high-frequency PA signals in liquid environments. It is worth mentioning that this device is also capable of capturing the vibrational spectrum of mesoscale particles up to hundreds of megahertz by contact. The microprobe device has the ability to be used in various complex scenarios beyond the laboratory while maintaining a high *Q* factor.

## Experiment

### Structure design and working principle

**Fig. 1:**
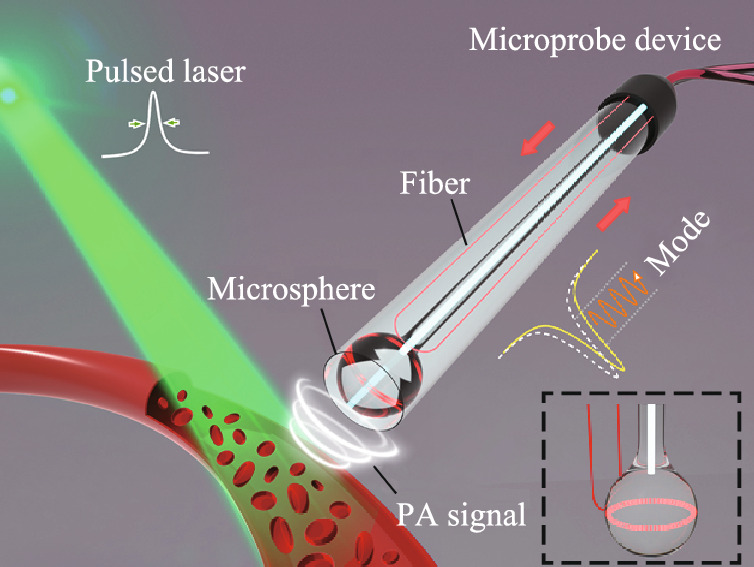
Structure of the microprobe device. It shows the coupling schematic (inset) and working principle of the microcavity mode for ultrasound signals

Here we propose a side-coupled method for the fiber-based microsphere, incorporating a folded tapered fiber as shown in Fig. 1. The U-shaped fiber is fabricated via hydrogen-flame tapering and precision axis-rotation bending, fixed by UV-cured adhesive for stability. The microsphere cavity is coupled to the tip of the folded tapered fiber which sends the pump light into the cavity and excites optical modes, as illustrated in the inset of Fig. 1. The fiber aligns with the microsphere stem direction, ensuring a compact overall structure. We employ a thin layer of low refractive index polymer droplets to encapsulate the coupling area, maintaining high ultrasound transmission efficiency. To further enhance the device’s durability, we reinforce it with a double-layer tube shell made of glass and aluminum alloy. In PA detection, the sample absorbs pulsed light and emits PA signal, which modulates the shape and refractive index of the microcavity. As a result, the microprobe device can effectively encode the ultrasound signal onto the optical mode with high sensitivity, enabling the extraction of amplitude and frequency information from the ultrasound signal through demodulation of the optical transmission signal.

### Experimental results and performance measurements

**Fig. 2:**
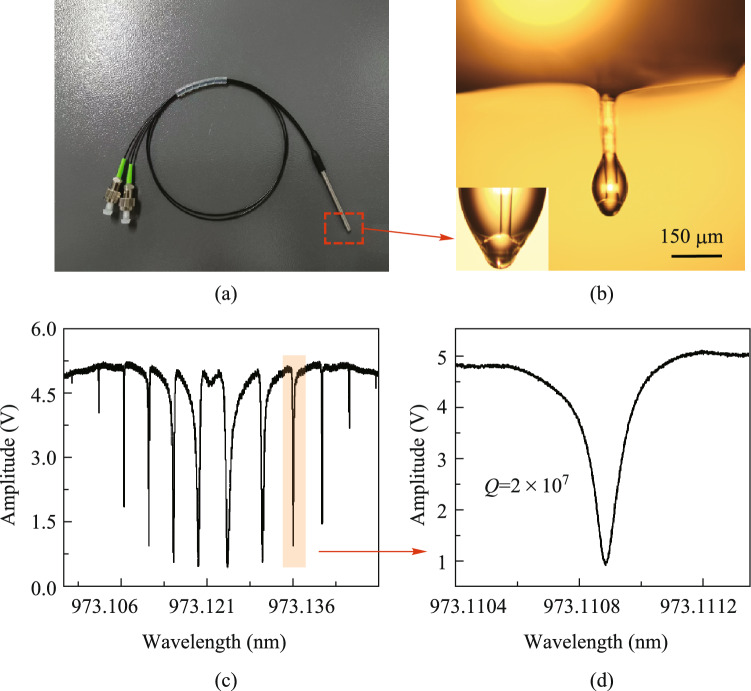
**a** Photograph of the microprobe device after packaging and ruggedization. **b** Enlarged view of the microprobe sensing unit. **c** Modes distribution of the microsphere tested by a tunable laser. **d** Enlarged view of one mode in **c**

Based on the proposed design, we successfully fabricated the microprobe device as shown in Fig. 2a. The microsphere sensing unit is located at the top of the tube shell. Standardized FC/APC port is implemented to enable plug-and-play utilization. The tube of aluminum alloy is just 2 mm. In Fig. 2b, the encapsulated structure of the sensing unit is displayed, with the microsphere measuring approximately 70 μm in size. The tapered fiber (diameter ~ 1 μm) is attached to the stem of the microsphere, ensuring an entire structure integrated. Characterization of the microcavity modes was conducted using a tunable laser (TOPICA, 910–980 nm), with the results presented in Fig. 2c. The analysis in Fig. 2d reveals an impressive maximum *Q* factor of 2 × 10^7^ for the microcavity. The compact dimensions and high *Q* of the microprobe facilitate an extremely high energy density of light for high sensitivity sensing. Furthermore, the integrated coupling structure enables versatile utilization of the device in both air and liquid environments.

**Fig. 3:**
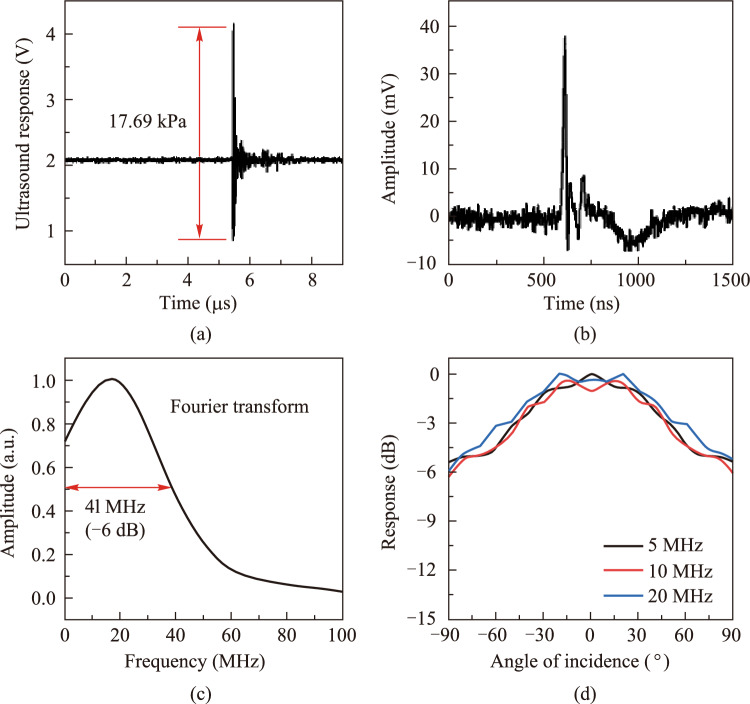
**a** Response of the microprobe device to the emitting signal of an ultrasound transducer. **b** Response of the microprobe device to a broadband PA signal. **c** Fourier transform of the photoacoustic response in **b**. **d** Response of the microprobe to the ultrasound at different incident angles

Further, we tested the ultrasound response of the microprobe device with results shown in Fig. 3. The sensitivity of our microprobe device is measured by detecting 20 MHz ultrasound waves emitted from an ultrasound transducer driven by a pulse/echo receiver. Under identical experimental conditions, the ultrasound signal was measured using an ONDA HGL-1000 hydrophone. For more information on the calibration process, please refer to our previous article [25]. The detected signal after a 1.2–800 MHz band-pass filter is illustrated in Fig. 3a. Notably, the noise equivalent pressure (NEP) is calculated to be as low as 24 Pa, or equally 5.4 mPa/√Hz. By comparison, the needle hydrophone exhibits an NEP of 768 Pa in established research [9]. This heightened sensitivity of our probe can be attributed to the exceptional *Q* factor of the microcavity. At the same time, the diminutive size of the cavity results in a significantly high energy density of pump light, which is also of benefit to sensitivity. To evaluate the frequency response of the device, we used a pulsed laser (pulse width ∼1.8 ns, pulse energy ∼2 μJ) to irradiate a copper film with a thickness of 50 nm to generate a broadband PA signal. The detected time-domain PA signal is shown in Fig. 3b, and its Fourier transform outcome is shown in Fig. 3c. The results indicate a broadband response exceeding 40 MHz in −6 dB, attributed to the cavity material’s acoustic properties (Young’s modulus and sound velocity) and the reflection properties of ultrasound in the cavity. This solution of integrating the cavity and the stem can make the ultrasound pass through the microcavity and be guided away, thus mitigating the occurrence of multiple sound waves reflections within the cavity. We also calibrated the response of the microprobe to ultrasound from different angles as shown in Fig. 3d. The response is within −6 dB over a range of 180°. As a point detector, the microsphere cavity can theoretically respond uniformly to the full space angle. However, the relative position of the protective shell to the microsphere may block some of the ultrasound. By adjusting the shell position, the receiving angle can be tailored as required.

### Photoacoustic imaging with microprobe device

We built the PA microscopy (PAM) imaging system which is basically the same as the system in our previous study [23, 24]. The pulsed light is focused by the objective lens and irradiates the sample from below. The microcavity probe captures the PA signal from above the sample during 2D scanning, with ultrasound amplitude extracted from time-domain pulse peaks after bandpass filtering. During imaging, both the sample and microprobe are immersed in an aqueous environment.

**Fig. 4:**
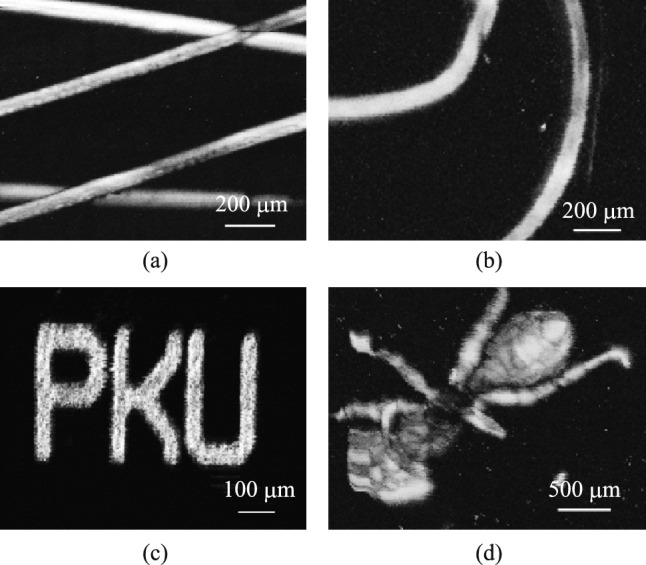
PA imaging of samples. **a** Tight and crossed hairs. **b** Curly hairs. **c** Letter-shaped gold film. **d** Ant

PAM was conducted on different samples, yielding compelling results. Figure 4a shows the PA image of hairs that are tightened and fixed to the glass slide. Not only the contours of the hair can be delineated in the PA image, but also the scaly structure on the surface of the hair. Figure 4b shows two curly hairs, buried in a hydrogel for PA imaging, still achieving high contrast. In addition, we performed PAM of a gold film engraved with letters in Fig. 4c. The width of the font is around 50 μm, displaying a clear outline edge. In Fig. 4d, we performed PAM of an ant that was immobilized in a hydrogel, showcasing a well-defined silhouette of the ant. However, one leg is not clearly displayed because it is bent and not in the focus of the pulsed light. These PAM examples demonstrate the exceptional performance and stability of our microprobe device. Each image takes no more than 15 min, and the optimal resolution was calibrated by scanning a blade edge: the transition signal's line spread function derivative yields a lateral resolution (FWHM), optimized to ~ 2 μm through NA and beam diameter adjustments. This resolution calibration protocol follows standardized methodologies widely adopted in photoacoustic imaging [24].

### Mesoscopic vibrational spectroscopy

Vibrational spectroscopy is a ubiquitous technology that derives the species, constituents and morphology of an object from its natural vibrations. Despite the challenges associated with the measurement of mesoscopic vibration spectroscopy, it presents new opportunities for the elucidation of various entities, including all kinds of particles [26, 27], as well as biological cells and viruses [28, 29]. Microcavity-based detector to conduct vibrational spectroscopy measurements of different bacteria and micro-nanoparticles is feasible in laboratory cleanrooms [19]. Here, our microprobe device cannot only be suitable for micro/nano particles’ broadband vibration spectroscopy measurements, but also hold significant promise for application and promotion beyond the laboratory. The microsphere is surrounded by a layer of polymer glue of a thickness of several microns, which will not impede the transmission of high-frequency ultrasound while fixing the coupling point. By focusing the 532 nm pulsed light on the particles adhering to the microprobe, the resultant broadband PA signal encompasses the entire vibrational spectrum of the particles. The signal of the mechanical spectrum is enhanced by resonance, and other frequencies are rapidly attenuated. The detected electrical signals were amplified (SHF 806E, 26 dB at 40 kHz–38 GHz) and recorded by an oscilloscope (Keysight, DSOS254A) with 64 times average.

**Fig. 5:**
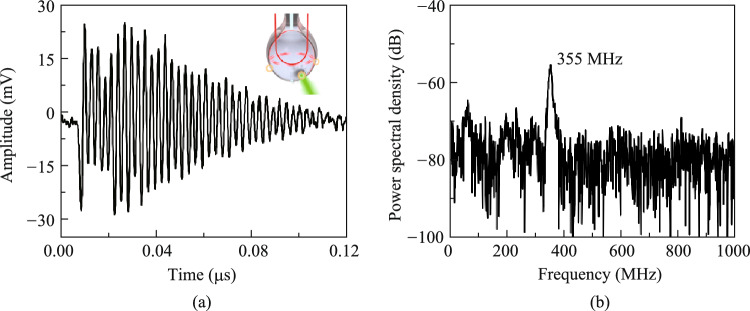
**a** Time-domain vibration signal from the polystyrene particles excited by pulsed light. The inset shows the schematic of measurement. **b** Vibrational spectrum in the frequency domain corresponding to **a**

The vibrational spectroscopy measurements of polystyrene particles with a radius about 2.8 μm are shown in Fig. 5. The diagram illustrates the time-domain signal of vibrations in (a) and its Fourier spectrum distribution is shown in (b). This particle exhibits vibrations exceeding 100 ns, with the main vibration peak located at 355 MHz. The measured frequency aligns with theoretical predictions [19] based on the natural frequency formula for elastic spheres: ${{\varvec{V}}}_{\left({\varvec{n}},{\varvec{l}}\right)}=\sqrt{{\varvec{E}}/{\varvec{\rho}}}\times {{\varvec{\xi}}}_{({\varvec{n}},{\varvec{l}})}/{\varvec{R}}$, given the material density of polystyrene is 1.05 g/cm^3^, the radius of polystyrene is 2.8 μm, Young’s modulus is 3.24 GPa, and Poisson ratio is 0.34. This method provides a novel avenue for the measurement of mesoscopic particle vibrational spectroscopy. It is essential to note that bandwidth measurement in PA imaging entails detecting of pulsed signals, while the vibrational spectroscopy involves continuous acoustic sensing. Therefore, the bandwidth of PA in −6 dB is different from the measurement range of the vibration spectrum. With sufficient sensitivity, the microcavity can measure vibrations well below −6 dB. The detection threshold of the WGM microcavity is governed by its *Q*-factor and operational frequency. Higher *Q* values not only extend the measurable frequency range but also enhance the sensitivity of sensing through intensified light-matter interactions. The upper limit frequency of our microprobe for vibrational spectroscopy measurements can exceed the GHz level [27].

## Discussion and conclusions

In this article, we have successfully demonstrated a WGM microprobe device with high *Q* factors. A novel coupling and encapsulation approach is proposed for ultrasensitive ultrasound detection and photoacoustic imaging. The coupling region is securely enclosed by a thin layer of low refractive index polymer, ensuring robust stability of the microcavity mode without compromising the detection of ultrasound. Our comprehensive evaluation encompassed the ultrasound response, the successful execution of photoacoustic imaging on diverse samples, and the measurement of vibration spectrum of mesoscopic objects with microcavity probes.

In experiments, the fabrication and coupling of folded tapered fibers are critical steps. The transmittance of the U-shaped fiber decrease during the coupling and encapsulation with the microcavity. This change in transmittance is mainly influenced by the following factors: (a) attenuation during the tapering process of the fiber from its original state; (b) attenuation during the folding of the tapered fiber into a U-shape; and (c) attenuation during the dispensing of adhesive and the encapsulation process. In coupling, a five-dimensional translation stage is required to precisely control the angle and the relative position of the microcavity to the fiber. In the experiments, we strictly monitor the transmittance changes during the U-shaped fiber preparation and coupling encapsulation process. When the encapsulation is fully completed, we can maintain a transmittance of more than 20%.

While we have highlighted two typical applications, the versatility of this microprobe can extend to various scenarios such as PA endoscopic imaging, gas/liquid PA spectroscopy, and metal flaw detection. Compared to microcavities on chips, our structure features a simpler package design, greater versatility in applications, and notable advantages in ultrasound response performance [16, 30]. This coupling structure offers considerable flexibility. By adjusting the size of the microsphere cavity, it can be applied to ultrasound measurements with different bandwidths and different center frequencies. It is applicable for ultrasound measurement in air as well as for detecting ultrasound signals in aqueous or other liquid environments. We firmly believe that the microprobe design proposed in this paper holds significant potential for applications in ultrasound detection and vibration measurement, promising advancements in various fields of high-sensitivity ultrasound sensing.

At the same time, we believe that this work provides a fully packaging solution, which has stability, high quality factor, and applicability to complex environments, which plays a great role in promoting the WGM microcavity from laboratory to industrial application. In addition, with the miniaturization and convenience of tuned lasers, microcavity systems can be used in a variety of applications beyond the laboratory.

## Data Availability

The data that support the findings of this study are available from the corresponding authors, upon reasonable request.
